# Serum and Urine Nerve Growth Factor and Glycosaminoglycan Levels in Obstructive and Non‐Obstructive Feline Urolithiasis and Interstitial Cystitis

**DOI:** 10.1002/vms3.70968

**Published:** 2026-05-20

**Authors:** Mehmet Maden, Ahmet Sarıkaya, Erdem Gülersoy, Hacer Marangoz, Zafer Sayın

**Affiliations:** ^1^ Department of Internal Medicine Veterinary Faculty Selçuk University Konya Türkiye; ^2^ Department of Internal Medicine Veterinary Faculty Harran University Şanlıurfa Türkiye; ^3^ Department of Microbiology Veterinary Faculty Selçuk University Konya Türkiye

**Keywords:** biomarker, cystitis, feline lower urinary tract disease, interstitial, obstruction, uroliths

## Abstract

**Background:**

Feline interstitial cystitis (FIC) results from complex interactions between the urinary bladder, nervous system, adrenal glands and the cat's living environment. Following FIC, urolithiasis is the second most common cause of feline lower urinary tract disease (FLUTD), often producing clinical signs that mimic those of obstructive FIC.

**Objectives:**

This study aimed to assess the diagnostic utility of serum and urine concentrations of nerve growth factor (NGF) and glycosaminoglycans (GAGs) in cats with urolithiasis or interstitial cystitis, under both obstructive and non‐obstructive conditions.

**Methods:**

The study included 90 cats, comprising 80 cats with clinical signs of FLUTD and 10 healthy controls. Group classification was based on clinical, laboratory and ultrasonographic examinations, as well as multimodal environmental modification (MEMO) evaluation criteria. Cats diagnosed with urolithiasis or FIC were further categorised into obstructive (urolithiasis/obstructive [URO/Ob], FIC/Ob) and non‐obstructive (urolithiasis/non‐obstructive [URO/Non‐Ob], FIC/Non‐Ob) subgroups.

**Results:**

Mild changes were noted in CBC and serum biochemistry. Serum and urine NGF levels were elevated in URO/Ob, FIC/Ob and FIC/Non‐Ob groups but not in URO/Non‐Ob. Serum and urine GAG levels were reduced in all groups except URO/Ob. In obstructive cases, serum (AUC = 0.693) and urine GAG (AUC = 0.629) showed moderate/acceptable diagnostic performance in distinguishing URO/Ob from FIC/Ob. In non‐obstructive cases, serum (AUC = 0.622) and urine NGF (AUC = 0.612) demonstrated limited to moderate ability to differentiate FIC/Non‐Ob from URO/Non‐Ob.

**Conclusions:**

Elevated serum and urine NGF concentrations in cats with FIC have been associated with increased severity of bladder inflammation and tissue injury. In contrast, reduced urine GAG concentrations, observed in all groups except the URO/Ob group, may facilitate bladder inflammation by increasing urothelial permeability. Accordingly, serum and urine GAG levels in obstructive cases and serum and urine NGF levels in non‐obstructive cases may provide clinically relevant insights into both the pathophysiology and diagnostic evaluation of FLUTD.

## Graphical abstract

Elevated NGF levels in serum and urine are associated with increased bladder inflammation and tissue damage in FIC cases. In contrast, reduced urinary GAG levels except in obstructive urolithiasis may contribute to bladder inflammation by enhancing urothelial permeability. Serum and urine GAG levels are more informative in obstructive FLUTD, whereas NGF levels provide better diagnostic insight in non‐obstructive forms, offering valuable biomarkers for differentiating FLUTD subtypes.

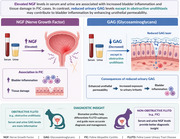

## 1 Introduction

Feline lower urinary tract disease (FLUTD), a term encompassing various lower urinary tract disorders in cats, is among the most common feline diseases, affecting approximately 1.5%–4.6% of cats presented to veterinary clinics (Forrester and Roudebush 2007; Lund et al. 2016; Westropp et al. 2019). In FLUTD cases, the reported distribution is approximately 55%–60% interstitial cystitis (IC), 12%–22% urolithiasis, 1.5%–20% bacterial urinary tract infections, 0.3%–3.6% bladder neoplasms and 0.2%–3% neurological disorders (Chew and Buffington 2013; Kim et al. 2018; Kaul et al. 2020).

Feline interstitial cystitis (FIC) is the most common and diagnostically challenging cause of FLUTD (Forrester and Towell 2015). Studies over the past two decades suggest that FIC arises from complex interactions among the urinary bladder, nervous system, adrenal glands and environmental factors (Mayer and Bushnell 2009; Forrester and Towell 2015). Urolithiasis, defined as the presence of urinary stones anywhere in the urinary tract (Gomes et al. 2018), is the most common cause of FLUTD after FIC, accounting for approximately 18% of cases, compared to 57.7% for FIC (Piyarungsri et al. 2020). Factors such as breed, gender, age, diet, obesity, home environment, climate and castration are considered important risk factors for the development of urolithiasis (Picavet et al. 2019; Lulich and Osborne 2009; Hesse et al. 2012). Urinalysis is a crucial component of the diagnostic evaluation for urolithiasis. Although crystalluria is an important finding, it is not a definitive indicator of urolith presence (Osborne et al. 2009). Urine specific gravity (Sg) and urine pH affect urolith formation and can provide information about the type of urolith (Bartges 2011). In cats diagnosed with FIC, common clinical signs include dysuria (77%), stranguria and periuria (70%), pollakiuria (78%) and macroscopic haematuria (71%; Defauw et al. 2011). However, these are non‐specific findings of FLUTD, and no test or clinical marker is currently specific for diagnosing FIC (Westropp et al. 2019; Gülersoy and Maden 2022). While imaging is crucial for detecting uroliths, serum biochemistry, especially electrolyte, mineral, creatinine (CRE) and blood urea nitrogen (BUN) levels, can provide valuable insights into the underlying causes and potential complications of urolithiasis (Savary et al. 2000). Due to the overlap in clinical signs among different FLUTD etiologies, the diagnostic process remains challenging and highlights the need for more specific diagnostic tests.

Given the time‐consuming nature of urine cultures, the limitations of urine test strips, and the inability of physical examinations to distinguish between FLUTD etiologies, recent studies have focused on elucidating the underlying pathogenesis and identifying potential diagnostic biomarkers (Dorsch et al. 2014; Mattoo and Spencer 2024). Altered concentrations of urinary and serum metabolites, including tryptophan, dopamine and kynurenine, have been reported in cats with FIC compared with healthy controls (Rubio‐Diaz et al. 2009; Gulersoy et al. 2022b). In addition, changes in circulating inflammatory and structural markers such as serum IL‐12 p40, CXCL12, IL‐18, Flt3 ligand (Parys et al. 2018), nerve growth factor (NGF) and glycosaminoglycans (GAGs; Gulersoy et al. 2022a, 2022b) have also been documented. NGF, produced by urothelial and smooth muscle cells, is a neurotrophin involved in sensory neuron development and modulation of bladder reflexes. In human IC/bladder pain syndrome (IC/BPS), increased NGF expression has been associated with altered pain perception and disease pathogenesis. Similarly, the GAG layer of the bladder urothelium functions as a protective barrier, and its disruption increases bladder permeability and promotes urothelial dysfunction in IC/BPS. Due to similarities with human IC/BPS, these mechanisms may also underlie FIC.

Given the high incidence of urolithiasis and its overlapping clinical signs with FIC, the primary aim of this study was to assess the diagnostic utility of serum and urine concentrations of NGF and GAGs in cats with these conditions, under both obstructive and non‐obstructive presentations. It was hypothesised that NGF and GAG concentrations would differ significantly between cats with urolithiasis and those with FIC in both clinical presentations and that ROC‐derived thresholds would provide reliable diagnostic discrimination between these groups.

## 2 Materials and Methods

### 2.1 Study Population

Between January 2023 and March 2024, 140 cats presenting with clinical signs suggestive of FLUTD were evaluated at Selçuk University Small Animal Hospital and affiliated private veterinary clinics in accordance with the study's inclusion and exclusion criteria. Based on an a priori sample size calculation performed using G*Power software (α = 0.05, power = 80%), a total of 90 cats (80 with FLUTD and 10 healthy controls) were ultimately enrolled to ensure adequate statistical power to detect significant differences in serum and urine biomarker concentrations among groups. All cats underwent anamnesis, physical examination, laboratory analyses (including urinalysis, urine culture, complete blood count [CBC] and serum biochemistry) and abdominal ultrasonography.

### 2.2 Inclusion/Exclusion Criteria

Cats that presented with lower urinary tract disease symptoms (dysuria, stranguria, haematuria, pollakiuria, periuria) were included in the FLUTD group of the study. Case selection was based on clinical evaluation, laboratory findings, abdominal ultrasonographic examination and multimodal environmental modification (MEMO) assessment criteria.

Cats with diseases detected in addition to or unrelated to FLUTD were excluded from the study. Exclusion criteria included evidence of renal or other systemic diseases based on clinical evaluation and serum biochemistry and urinalysis; ultrasonographic findings such as organomegaly, altered parenchymal echogenicity, abnormal masses or free abdominal fluid; and the presence of abnormal formations (e.g., tumours) within the urinary bladder. Additionally, cats with positive bacterial cultures from urine samples obtained by cystocentesis were excluded. Cases with comorbid conditions including endocrinopathies (e.g., hyperthyroidism), pyelonephritis, chronic kidney disease, urolithiasis or those receiving medications that could influence urinary or inflammatory parameters (such as antibiotics, antihistamines, corticosteroids, nonsteroidal anti‐inflammatory drugs, anticholinergics, antidepressants, urinary acidifiers, GAGs, diuretics or other treatments commonly used for FIC) were also excluded.

### 2.3 Clinical Evaluation and MEMO Scoring

All clinical examinations were performed in a dedicated feline examination room by the same evaluator, using minimal restraint to minimise stress and avoid potential influence on the study outcomes. The clinical examination included measurements of rectal body temperature, respiratory and heart rates, as well as lung and heart auscultation. Palpation of the mandibular, prescapular, superficial inguinal and popliteal lymph nodes was also performed. Physical examination parameters were evaluated solely for inclusion or exclusion purposes in this study.

Following physical examination and confirmation of eligibility, cats whose owners consented to participation were enrolled in the MEMO study. Informed consent for participation in the MEMO study was obtained from the owners of cats that met the inclusion criteria. An interview with the client was conducted either in person or by telephone to obtain a review of systems and environmental history (Westropp and Buffington 2004). The history included a standardised questionnaire that each client completed in its entirety. MEMO scoring was performed by a single evaluator who was blinded to the clinical diagnosis and group allocation of the cats. Questions were asked about FLUTD and a variety of clinical signs pertaining to various other organ systems, and about the cat's current environment. The MEMO score was determined between 0 and 5, according to the responses (0 = never, 1 = once ever, 2 = once a year, 3 = once a month, 4 = once a week, 5 = everyday; Buffington et al. 2006). MEMO scores, assessed at admission, were compared between groups using the Kruskal–Wallis test followed by Dunn's post hoc test. The MEMO score was used solely as an inclusion criterion to document exposure to stress‐related factors and to ensure comparable clinical context among cats. As no validated cut‐off value exists for the MEMO score, inclusion was based on the identification of stressors through this assessment rather than on a predefined numerical threshold. Therefore, correlation analyses between MEMO scores and biomarker concentrations were not performed, as this was beyond the scope of the study objectives.

### 2.4 Forming Subgroups

Based on the MEMO evaluation (Buffington et al. 2006), clinical and laboratory examinations, urine bacterial culture results and the presence of uroliths, the cats with FLUTD were divided into two groups: URO (*n* = 54, including 19 intact males, 27 neutered males; three intact females, five neutered females) and FIC (*n* = 26, including five intact males, 11 neutered males; four intact females, six neutered females). FLUTD cases were further categorised into obstructive urolithiasis (URO/Ob), non‐obstructive urolithiasis (URO/Non‐Ob), obstructive FIC (FIC/Ob) and non‐obstructive FIC (FIC/Non‐Ob). Cats with normal findings on physical examination, laboratory testing and ultrasonographic evaluation were included in the control group.

### 2.5 Sample Collection and Laboratory Examination

Venous blood samples (collected in K_3_EDTA tubes for anticoagulation and gel serum tubes without anticoagulant) and urine samples were obtained from all cats by trained veterinary staff using appropriate, stress‐minimising methods as previously described (Gulersoy et al. 2022a, 2022b). After centrifugation of blood and urine samples (10 min at 5000 rpm), the supernatant was transferred to Eppendorf tubes within approximately 60 min and stored at −20°C. Samples were aliquoted at the time of initial processing, and each aliquot underwent only a single freeze–thaw cycle prior to ELISA measurement to prevent degradation of NGF and GAGs. All analyses were performed within four months of collection (Gulersoy et al. 2022a). Venous blood samples collected in K_3_EDTA tubes were analysed immediately after collection in the hospital's central laboratory for CBC parameters; therefore, no storage or freeze–thaw cycles were involved. The CBC profile included leukocyte (WBC), haemoglobin (Hb), haematocrit (Ht), erythrocyte (RBC), platelet count (PLT), mean corpuscular volume (MCV), mean corpuscular Hb (MCH), mean corpuscular Hb concentration (MCHC), lymphocytes, monocytes and granulocytes. Serum samples obtained from plain tubes were likewise processed and analysed immediately in the same laboratory for biochemistry profiling, including BUN, CRE, glucose (GLU), total protein (TP), albumin (ALB), alanine aminotransferase (ALT), alkaline phosphatase (ALP), cholesterol (TCHO), triglycerides (TG), calcium (Ca) and phosphorus (P), with no storage or freeze–thaw cycles applied.

### 2.6 Ultrasound Protocol and Urinalysis

All ultrasonographic examinations were performed by a single experienced operator who was blinded to the cats’ group allocations and clinical data. During abdominal palpation before ultrasonographic examination, some cats with URO or FIC showed no bladder distension. To avoid dilution or alteration of biomarker concentrations, urine samples were collected prior to intravesical saline administration (4 mL/kg) used to standardise bladder ultrasonography, as previously recommended (Gulersoy et al. 2022a). This procedure achieved moderate bladder distension in all cats. Ultrasonographic examination was performed in ventrodorsal recumbency using a 5–7.5 MHz convex probe (Mindray Z60, China) by an expert operator blinded to the clinical status. Urine dipstick and sediment examinations were conducted within 30 min of collecting urine samples via cytocentesis. For the sediment examination, 4–5 mL of urine was centrifuged at 1500 rpm for 5 min. The supernatant was discarded, and the remaining urine sediment was resuspended and examined by light microscopy at ×100 magnification (Olympus CX21, Japan).

The presence of RBC, WBC, bacteria, crystals and epithelial cells in the urine sediment was evaluated. Haematuria was defined as RBC >10 per microscopic field (HPF), and pyuria was defined as WBC > 5 per field. The density of crystals and cells was graded as mild (+), moderate (++) or severe (+++) based on their quantity. Urine density was measured using a refractometer. Dipstick analyses assessed pH, protein, RBC, WBC and the presence of crystals (Gulersoy et al. 2022b).

For urine culture, urine samples were diluted 10‐fold to a final concentration of 1 × 10^−^^8^ using physiological saline. A 0.1 mL aliquot of the diluted samples was inoculated onto blood agar with 5% sheep blood (Oxoid), MacConkey agar (Oxoid) and Columbia CNA agar (Oxoid) plates. The plates were incubated anaerobically at 37°C and examined at 18, 24 and 48 h. Significant bacteriuria was defined as bacterial counts >10^5^ colony‐forming units (CFUs) per milliliter (Bartges 2004).

### 2.7 Biomarker Analysis

Serum and urine samples used for ELISA were coded numerically, and the laboratory personnel conducting the assays were blinded to the clinical data and diagnoses until all analyses were completed. Inflammatory and damage biomarkers; NGF (detection range: 6.25–200 ng/mL, sensitivity: 1.0 ng/mL, Assay Type: Quantitative Sandwich, Catalogue No.: MBS093622) and GAG (detection range: 0.5–200 ng/mL, sensitivity: 0.31 ng/mL, Assay Type: Quantitative Sandwich, Catalogue No.: MBS1608261), were measured in serum and urine supernatant using feline‐specific quantitative colorimetric sandwich ELISA kits, according to the manufacturer's instructions (Bioassay Technology Laboratory, Zhejiang, China).

### 2.8 Statistical Methods

The Shapiro–Wilk test was used to assess the normality of the data. Parametric data were analysed using the Student's *t*‐test and are presented as mean ± standard deviation (SD). A post hoc power analysis indicated that the study had > 80% power to detect moderate‐to‐large effect sizes (Cohen's *d* > 0.7) in biomarker levels between groups, supporting the validity of the main comparisons. Data from serum and urine samples of healthy and FLUTD‐affected cats were analysed using one‐way ANOVA for multiple independent groups, with post hoc Tukey's test for pairwise comparisons. Receiver operating characteristic (ROC) curve analysis was used to determine diagnostic cut‐off values for evaluating the clinical efficacy of NGF and GAG in serum and urine. The analysis also assessed their ability to differentiate between URO and FIC based on the condition (obstructive or non‐obstructive). The clinical performance of biomarkers is evaluated with parameters, including the area under the curve (AUC > 0.600), *p*‐value (< 0.05), sensitivity and specificity (> 70%). To assess the test's ability to discriminate between URO and FIC, or between obstructive and non‐obstructive conditions, threshold values for ROC parameters were interpreted as follows: an AUC of 0.5 indicates no discrimination, 0.6–0.8 is considered moderate/acceptable, 0.8–0.9 excellent and > 0.9 outstanding (Hosmer and Lemeshow 2000). However, in the present study, these statistical classifications do not necessarily translate into clinical utility; therefore, the diagnostic performance of the biomarkers was interpreted cautiously in a clinical context. Statistical significance was considered as *p* < 0.05. All analyses were performed using SPSS 27.00 statistical software program.

## 3 Results

### 3.1 Study Population

All cats were between 1 and 7 years of age and represented various breeds and both sexes. In the FLUTD group, there were 24 intact males, 38 castrated males, seven intact females and 11 spayed females, whereas the control group comprised three intact males, four castrated males and three intact females. All cats were indoor‐only pets, fed commercial dry diets, housed without other pets and received routine vaccinations and regular deworming.

### 3.2 Clinical Evaluation Findings

**FIGURE 1:**
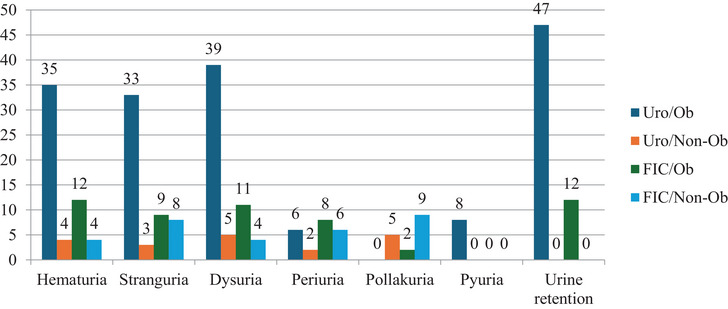
Distribution of clinical examination findings of the affected cats.

Clinical signs such as haematuria, stranguria, dysuria and urinary retention were most frequently observed in the URO/Ob group. Haematuria, stranguria, dysuria, periuria, pollakiuria, pyuria and urinary retention were identified based on anamnesis and physical examination findings as illustrated in Figure 1.

**FIGURE 2:**
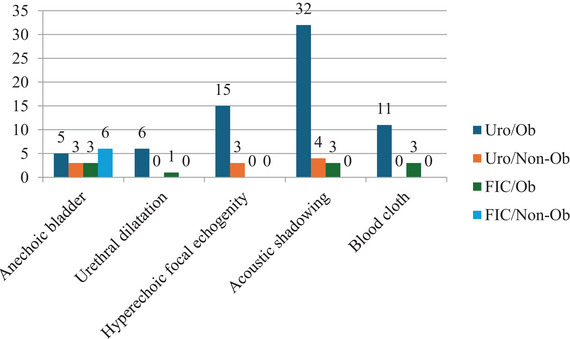
Distribution of USG examination findings of the affected cats.

Ultrasonographic examination revealed an anechoic urinary bladder, urethral dilatation, focal hyperechoic structures with acoustic shadowing and sedimented echogenic material consistent with blood clots as illustrated in Figure 2. Additionally, ultrasonographic examination revealed significant bladder wall thickening (> 0.2 cm) in cats with FLUTD, consistent with previous studies (Gulersoy et al. 2022a; Gülersoy and Maden 2022). Mean bladder wall thickness measurements were as follows: URO/Ob: 0.25 ± 0.05 cm, URO/Non‐Ob: 0.23 ± 0.02 cm, FIC/Ob: 0.24 ± 0.03 cm and FIC/Non‐Ob: 0.25 ± 0.05 cm. In contrast, the control group exhibited a mean thickness of 0.14 cm. However, no statistically significant difference was observed between the FLUTD groups (*p* > 0.840).

### 3.3 Laboratory Examination Findings

#### 3.3.1 CBC and Serum Biochemistry

Mild changes were observed in CBC and serum biochemistry analyses. Hb concentrations in the URO/Non‐Ob group (*p* < 0.02), as well as ALB (*p* < 0.036) and total cholesterol (*p* < 0.048) concentrations, were higher than in the control group. All remaining parameters were within the reference intervals. CBC findings were presented in Table 1, and serum biochemistry profiling was presented in Table 2.

**TABLE 1:** CBC findings.

Parameters	URO/Ob (*n*: 47) mean ± SD	URO/Non‐Ob (*n*: 7) mean ± SD	FIC/Ob (*n*: 12) mean ± SD	FIC/Non‐Ob (*n*: 14) mean ± SD	Control (*n*: 10) mean ± SD	*p*‐value
WBC (m/mm^3^)	12.45 ± 0.79	10.35 ± 1.99	15.35 ± 2.29	11.78 ± 1.37	9.80 ± 1.10	0.175
NEU (m/mm^3^)	8.41 ± 0.73	7.07 ± 1.44	10.85 ± 2.35	8.34 ± 1.10	5.92 ± 0.91	0.25
LYM (m/mm^3^)	2.86 ± 0.24	2.77 ± 0.86	3.64 ± 0.52	2.86 ± 0.55	3.21 ± 0.43	0.70
MON (m/mm^3^)	0.16 ± 0.02	0.18 ± 0.12	0.27 ± 0.05	0.18 ± 0.03	0.11 ± 0.01	0.29
EOS (m/mm^3^)	0.42 ± 0.04	0.29 ± 0.12	0.58 ± 0.08	0.38 ± 0.08	0.54 ± 0.09	0.17
BAS (m/mm^3^)	0.00 ± 0.00	0.00 ± 0.00	0.00 ± 0.00	0.00 ± 0.00	0.00 ± 0.00	0.95
RBC (m/mm^3^)	8.71 ± 0.24	8.06 ± 0.75	16.05 ± 7.91	8.65 ± 0.41	8.75 ± 0.33	0.23
HB (g/dL)	**12.82 ± 0.23^a^**	**21.82 ± 9.56^b^**	**11.38 ± 0.69^a^**	**12.14 ± 0.53^a^**	**12.49 ± 0.41^a^**	**0.02**
HCT (%)	35.67 ± 0.65	32.40 ± 2.88	31.40 ± 1.75	34.00 ± 1.44	35.00 ± 0.87	0.08
MCV (fL)	39.36 ± 0.89	40.35 ± 1.03	37.81 ± 0.72	39.48 ± 0.74	40.36 ± 1.40	0.76
MCHC (g/dL)	357.87 ± 2.00	360.14 ± 5.32	362.66 ± 3.16	357.57 ± 2.84	356.80 ± 8.22	0.86
RDW‐CV (%)	**18.04 ± 0.25^ab^**	**18.25 ± 1.09^ab^**	**19.79 ± 0.54^a^**	**17.78 ± 0.38^c^**	**19.57 ± 0.54^a^**	**0.008**
RDW‐SD (fL)	30.12 ± 0.60	30.85 ± 2.70	31.34 ± 1.08	29.44 ± 0.81	33.17 ± 1.95	0.29
PLT (m/mm^3^)	220.17 ± 16.77	216.71 ± 32.91	232.41 ± 39.85	223.50 ± 20.17	191.60 ± 30.31	0.93
MPV (fL)	12.42 ± 0.20	11.31 ± 0.25	11.57 ± 0.42	12.50 ± 0.27	12.66 ± 0.32	0.06
PCT (mL/L)	2.67 ± 0.19	2.43 ± 0.34	2.88 ± 0.38	2.77 ± 0.23	2.40 ± 0.37	0.87

Abbreviations: BAS, basophil; EOS, eosinophil; Hb, haemoglobin; HCT, haematocrit; LYM, lymphocyte; MCHC, mean corpuscular haemoglobin concentration; MCV, mean corpuscular volume; MON, monocyte; MPV; mean platelet volume; NEU, neutrophil; PLT, platelet; RBC, erythrocyte; RDW, reticulocyte distribution width; WBC, leukocyte. Bold values indicate statistically significant differences between groups (*p* < 0.05).

**TABLE 2:** Serum biochemistry profiling findings.

Parameters	URO/Ob (*n*: 47) mean ± SD	URO/Non‐Ob (*n*: 7) mean ± SD	FIC/Ob (*n*: 12) mean ± SD	FIC/Non‐Ob (*n*: 14) mean ± SD	Control (*n*: 10) mean ± SD	*p*‐value
TP (g/dL)	8.05 ± 1.47	6.78 ± 0.18	11.85 ± 4.96	6.62 ± 0.18	6.58 ± 0.19	0.648
ALB (g/dL)	**3.07 ± 0.07^ab^**	**3.17 ± 0.19^ab^**	**3.56 ± 0.13^b^**	**3.29 ± 0.13^ab^**	**2.97 ± 0.17^a^**	**0.036**
ALP (U/L)	32.74 ± 1.69	26.57 ± 3.10	34.00 ± 2.39	32.57 ± 2.10	29.40 ± 3.46	0.521
GLU (mg/dL)	115.04 ± 3.54	107.57 ± 5.30	111.00 ± 2.67	116.64 ± 10.16	115.30 ± 5.88	0.920
TBIL (mg/dL)	0.28 ± 0.01	0.27 ± 0.03	0.26 ± 0.02	0.27 ± 0.02	0.27 ± 0.03	0.937
IP (mg/dL)	4.47 ± 0.09	4.50 ± 0.24	4.35 ± 0.21	4.40 ± 0.16	3.93 ± 0.37	0.333
TCHO (mg/dL)	**156.72 ± 3.26**	**143.57 ± 7.13**	**147.08 ± 6.58**	**155.85 ± 5.49**	**136.50 ± 3.94**	**0.048**
GPT (U/L)	63.97 ± 2.32	59.60 ± 5.5	62.75 ± 2.83	66.92 ± 5.37	56.70 ± 4.06	0.602
Ca (mg/dL)	10.00 ± 0.11	9.92 ± 0.34	9.61 ± 0.29	10.00 ± 0.19	9.63 ± 0.26	0.499
CRE (mg/dL)	1.18 ± 0.03	1.18 ± 0.07	1.20 ± 0.05	1.19 ± 0.05	1.23 ± 0.08	0.983
BUN (mg/dL)	27.08 ± 0.92	25.92 ± 2.83	23.52 ± 1.81	26.48 ± 1.79	24.57 ± 0.64	0.419

Abbreviations: ALB, albumin; ALP, alkaline phosphatase; BUN, blood urea nitrogen; Ca, calcium; CRE, creatine; GLU, glucose; GPT, glutamic pyruvic transaminase; IP, phosphorous; TBIL, total bilirubin; TCHO, total cholesterol; TP, total protein. Bold values indicate statistically significant differences between groups (*p* < 0.05).

### 3.4 Urinalysis, Sediment Examination and Urine Culture Findings

In cats with URO and FIC, urine WBC counts were significantly higher in the URO/Non‐Ob group, compared with the control and other groups (*p* < 0.001). In contrast, urine RBC counts were significantly increased in the URO/Ob and FIC/Ob groups, compared with the control and non‐obstructive groups (*p* < 0.001). There was no significant difference in urine Sg, pH and protein levels between the groups. Crystalluria was observed at mild (12/47), moderate (10/47) and severe (25/47) levels in the URO/Ob group; at mild (3/7) and moderate (4/7) levels in the URO/Non‐Ob group; and at mild levels (3/12) in the FIC/Ob group. No bacterial growth was detected in urine cultures from cats in the URO, FIC and control groups.

### 3.5 MEMO Scoring Findings

The prevalence of MEMO findings was analysed using monthly (Score 3), weekly (Score 4) and daily (Score 5) frequency scores across the URO/Ob, URO/Non‐Ob, FIC/Ob, FIC/Non‐Ob and control groups. Reported findings were categorised into lower urinary tract signs, behavioural signs and non‐specific clinical signs.

Non‐specific clinical signs—such as loss of appetite, reduced food and water intake, decreased general activity and vomiting—along with lower urinary tract signs, including stranguria and haematuria, were more frequently observed in obstructive cases (URO/Ob and FIC/Ob; see Figure S3). Periuria was more commonly reported in the FIC groups, whereas pollakiuria was more prevalent in the non‐obstructive groups (URO/Non‐Ob and FIC/Non‐Ob). Behavioural signs, including fear, anxiety, tension, aggression, excessive grooming or licking and reduced play and social interaction, were more pronounced in the URO/Ob, FIC/Ob and FIC/Non‐Ob groups. Urine spraying was significantly more common in the FIC/Non‐Ob group, compared to the others. Total MEMO scores were markedly higher in all FLUTD groups, with median scores of 28.27 (range: 8–54) in URO and 27.46 (range: 15–52) in FIC, compared to 2.6 (range: 1–4) in the control group.

### 3.6 Biomarker Analysis Findings

In comparing serum and urine NGF and GAG concentrations between healthy cats and those with FLUTD, the following results were observed. Serum NGF (*p* < 0.000) and urine NGF (*p* < 0.002) concentrations were higher in the URO/Ob (61.82 ± 18.36 and 57.71 ± 18.55, respectively), FIC/Ob (60.46 ± 19.13 and 55.86 ± 17.31, respectively), and FIC/Non‐Ob (67.92 ± 16.45 and 63.79 ± 15.68, respectively) groups but not in the URO/Non‐Ob group (59.19 ± 21.46 and 54.02 ± 21.37, respectively). Serum GAG (*p* < 0.005) and urine GAG (*p* < 0.005) concentrations were lower in all groups except the URO/Ob group (61.69 ± 20.40 and 56.01 ± 20.92, respectively). A summary of biomarker concentrations is provided in Table 3.

**TABLE 3:** Biomarker analysis results.

Parameters	URO/Ob (*n*: 47) mean ± SD	URO/Non‐Ob (*n*: 7) mean ± SD	FIC/Ob (*n*: 12) mean ± SD	FIC/Non‐Ob (*n*: 14) mean ± SD	Control (*n*: 10) mean ± SD	*p*‐value
NGF (urine) (ng/mL)	57.71 ± 18.55^A^	54.02 ± 21.37^AB^	55.86 ± 17.31^A^	63.79 ± 15.68^A^	34.52 ± 0.74^B^	0.002
NGF (serum) (ng/mL)	61.82 ± 18.36^A^	59.19 ± 21.46^AB^	60.46 ± 19.13^A^	67.92 ± 16.45^A^	40.44 ± 0.87^B^	0.0001
GAG (urine) (ng/L)	56.01 ± 20.92^AB^	42.18 ± 18.64^B^	47.55 ± 16.66^B^	41.17 ± 20.69^B^	76.72 ± 12.05^A^	0.005
GAG (serum) (ng/mL)	61.69 ± 20.40^AB^	44.60 ± 20.70^B^	48.03 ± 17.54^B^	50.05 ± 17.08^B^	79.33 ± 13.11^A^	0.0001

*Note*: A, B: AB test was used to explore the relationship between two datasets. Statistical difference was observed between A and B but no difference between A and AB and B and AB (*p* < 0.05; Kruskal–Wallis test).

Abbreviations: FIC, feline interstitial cystitis; GAG, glycosaminoglycan; NGF, nerve growth factor; Non‐Ob, non‐obstructive; Ob: obstructive, URO, urolithiasis.

### 3.7 Evaluation of ROC Curves for Serum and Urine Biomarkers

**FIGURE 3:**
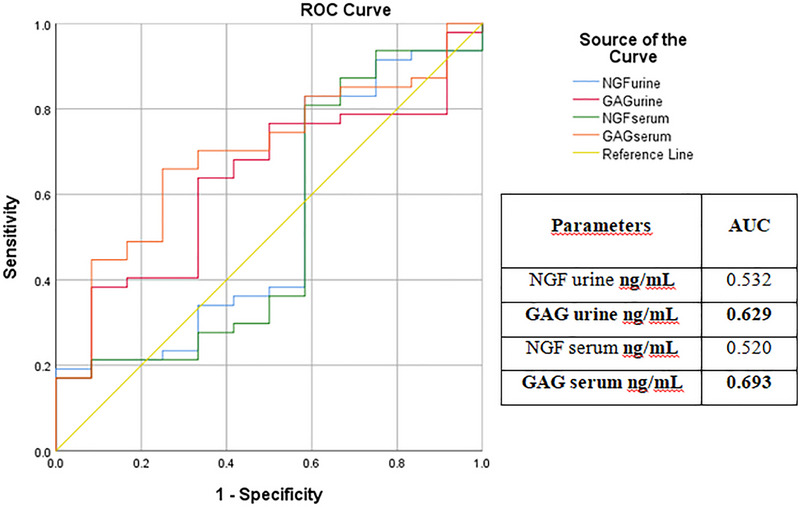
ROC curve analysis of serum and urine NGF and GAG concentrations for differentiating URO/Ob and FIC/Ob groups. The AUC values were 0.520 (serum NGF), 0.532 (urine NGF), 0.693 (serum GAG) and 0.629 (urine GAG), indicating moderate diagnostic performance. Bold values indicate statistically significant differences between groups (*p* < 0.05).

**FIGURE 4:**
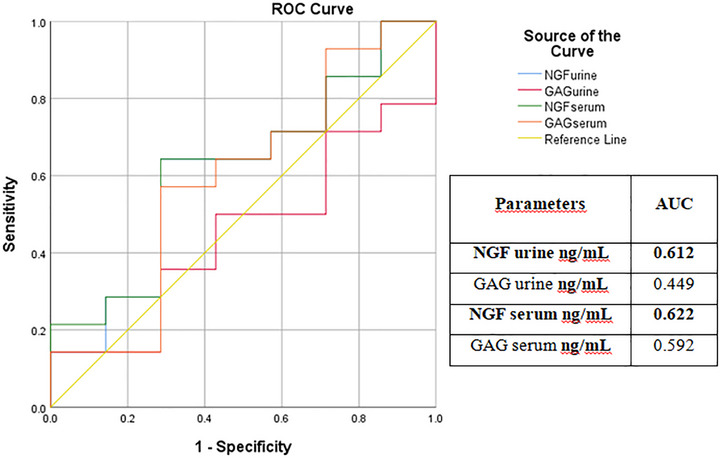
ROC curve analysis of serum and urine NGF and GAG concentrations for differentiating FIC/Non‐Ob versus URO/Non‐Ob groups. The AUC values were 0.622 (serum NGF), 0.612 (urine NGF), 0.592 (serum GAG) and 0.449 (urine GAG), indicating limited to moderate diagnostic performance. Bold values indicate statistically significant differences between groups (*p* < 0.05).

In obstructive conditions, serum GAG (AUC = 0.693; optimal cut‐off = 42.18, with 83% sensitivity and 41.7% specificity) and urine GAG (AUC = 0.629; optimal cut‐off = 42.67, with 76.6% sensitivity and 50% specificity) demonstrated better clinical performance than NGF in distinguishing URO/Ob from FIC/Ob cases (Table 2). Conversely, in non‐obstructive conditions, serum NGF (AUC = 0.622; optimal cut‐off = 66.54, with 64.3% sensitivity and 71.4% specificity) and urine NGF (AUC = 0.612; optimal cut‐off = 62.73, with 64.3% sensitivity and 71.4% specificity) were more effective than GAG in differentiating FIC/Non‐Ob from URO/Non‐Ob cases. ROC analysis results are presented in Tables 4 and 5, with ROC curves shown in Figures 3 and 4.

**TABLE 4:** ROC analysis results of obstructive cases (URO/Ob vs. FIC/Ob).

Parameters	AUC	Std. er.	*p*‐value	Asymp. %95 CI		Cut‐off	Sensitivity	Specifity
				Lower bound	Upper bound			
NGF urine (ng/mL)	0.532	0.099	0.735	0.338	0.726	43.45	83%	41.7%
GAG urine (ng/mL)	**0.629**	**0.082**	**0.169**	**0.469**	**0.790**	**42.67**	**76.6%**	**50%**
NGF serum (ng/mL)	0.520	0.103	0.836	0.318	0.721	47.86	80.9%	41.7%
GAG serum (ng/mL)	**0.693**	**0.078**	**0.040**	**0.540**	**0.847**	**42.18**	**83%**	**41.7%**

Abbreviations: AUC, area under curve; CI, confidence interval; GAG, glycosaminoglycan; NGF, nerve growth factor; Std. er, standard error. Bold values indicate statistically significant differences between groups (*p* < 0.05).

**TABLE 5:** ROC analysis results of non‐obstruction cases (FIC/Non‐Ob vs. URO/Non‐Ob).

Parameters	AUC	Std. er.	*p*‐value	Asymp. %95 CI		Cut‐off	Sensitivity	Specifity
				Lower bound	Upper bound			
NGF urine (ng/mL)	**0.612**	**0.134**	**0.412**	**0.350**	**0.875**	**62.73**	**64.3%**	**71.4%**
GAG urine (ng/mL)	0.449	0.130	0.709	0.194	0.704	40.60	50%	57.1%
NGF serum (ng/mL)	**0.622**	**0.131**	**0.371**	**0.366**	**0.879**	**66.54**	**64.3%**	**71.4%**
GAG serum (ng/mL)	0.592	0.141	0.502	0.316	0.868	42.13	64.3%	57.1%

Abbreviations: AUC, area under curve; CI, confidence interval; GAG, glycosaminoglycan; NGF, nerve growth factor; Std. er, standard error. Bold values indicate statistically significant differences between groups (*p* < 0.05).

## 4 Discussion

This study investigated the impact of urinary tract obstruction on bladder inflammation and tissue damage in cats with FIC or urolithiasis. Serum and urine concentrations of NGF and GAG were assessed, and their diagnostic sensitivity and specificity were evaluated. Comparative analyses revealed significant differences in serum and urine NGF and GAG concentrations among most groups, except for serum NGF in the URO/Non‐Ob group and urinary GAG in the URO/Ob group. These findings suggest that both biomarkers reflect bladder inflammation and tissue injury, largely independent of obstruction status. ROC analyses demonstrated that, in obstructive cases, urine GAG concentrations had greater diagnostic value than NGF in differentiating URO/Ob from FIC/Ob cases, highlighting GAG's clinical relevance as an indicator of urothelial barrier integrity and mucosal damage, features that are important for prognosis and therapeutic decision‐making in obstructive disease. Conversely, in non‐obstructive cases, serum and urine NGF concentrations outperformed GAG in distinguishing FIC/Non‐Ob from URO/Non‐Ob cases, supporting a stronger association between FIC and neurogenic bladder inflammation. Overall, serum and urine NGF may serve as useful markers of bladder inflammation and aid in differentiating FIC from urolithiasis in non‐obstructive presentations. Clinically, these findings suggest that incorporating NGF and GAG measurements into routine evaluation may improve diagnostic stratification and support more targeted management of cats presenting with FLUTD.

FLUTD is commonly associated with clinical signs such as stranguria, pollakiuria, haematuria and periuria (Forrester and Towell 2015). In cats with FIC, these manifestations may be accompanied by stress‐related behavioural changes, including anxiety, aggression, fear and increased sensitivity to environmental stimuli (Stella et al. 2011). In a previous study of 185 cats with FLUTD, stranguria (45.3%) and pollakiuria (11.9%) were the most frequently reported signs (Nururrozi et al. 2020). In the present study, dysuria (73.75%), haematuria (68.75%), stranguria (66.25%), periuria (27.5%) and pollakiuria (17.5%) were the most commonly observed clinical findings among 80 cats with FLUTD, with these signs occurring predominantly in obstructive cases, particularly within the URO/Ob and FIC/Ob groups. Specifically, stranguria was common in both obstructive and non‐obstructive FIC cases, while dysuria was more prevalent in obstructive URO and FIC groups. Pyuria occurred more frequently in URO/Ob cats. The severity and distribution of these clinical findings are likely influenced by the degree and duration of obstruction, as well as the extent of urinary tract inflammation (Jones et al. 2021). In the present study, these pathophysiological processes were also reflected in altered GAG and NGF concentrations as discussed below. In contrast to obstructive cases, periuria was more frequently reported in the FIC groups, possibly reflecting stress‐related behavioural disturbances such as urine marking, altered elimination patterns and increased attachment to owners (Barcelos et al. 2018; Jones et al. 2021). Pollakiuria was more commonly observed in non‐obstructive presentations. Collectively, these findings suggest that the severity and pattern of clinical signs in FLUTD are closely associated with the degree of underlying bladder inflammation and tissue damage (Dinler Ay et al. 2021).

The pathophysiology of FIC is thought to involve dysregulation of the neuroendocrine axis, often triggered by environmental stressors. To address this, MEMO strategies have been recommended to help reduce stress‐related neuroendocrine activation and improve clinical outcomes in affected cats (Buffington et al. 2006). In the present study, behavioural signs suggestive of stress including fear, anxiety, aggression, increased grooming or licking, reduced play and social interaction and urine spraying were observed more frequently in all FIC groups, compared to the URO group. These findings support the hypothesis that stress plays a central role in the development and clinical manifestation of FIC (Jones et al. 2021). Behavioural complaints such as vocalisation, whining, crying, hiding and withdrawal observed across all groups were considered potentially indicative of pain (He et al. 2022). The clinical presentation of cats with urolithiasis may vary depending on the location, number and composition of uroliths (Lulich and Osborne 2009). In particular, non‐specific signs such as vomiting and anorexia, or even an absence of overt clinical signs, may be observed when calculi are located in the upper urinary tract (Osborne et al. 2009). In this study, higher MEMO systemic sign scores observed in obstructed cats were likely associated with irritation and tissue damage caused by uroliths. Moreover, cats with urolithiasis exhibited higher overall MEMO scores than those with FIC, regardless of obstruction status, suggesting a greater stress‐related burden associated with the presence of uroliths. Living in multi‐cat households has previously been identified as a significant risk factor for the development of FLUTD (Lund et al. 2016; Kim et al. 2018). Consistent with these findings, the introduction of a new cat into the household was associated with an increase in FLUTD‐related clinical signs in approximately half of the cats in the present study (41/80, 51.25%). Based on MEMO scoring and anamnestic evaluation, these cats had previously been housed as single pets, suggesting that household change and increased competition may have contributed to stress‐related disease exacerbation. Collectively, these findings support the view that multi‐cat environments and household changes represent important risk factors for the development and exacerbation of FLUTD (Defauw et al. 2011; Lund et al. 2016; Kim et al. 2018).

In cases of FLUTD, haematology and serum biochemistry analyses are crucial for excluding concurrent diseases and other conditions that may cause FLUTD symptoms. Typically, routine haematology and serum biochemistry results in cats with FIC are normal (He et al. 2022). Diagnostic tests, including haematology and imaging, are also valuable in cases of urolithiasis. Additionally, in cases with obstruction, it is important to monitor changes such as azotemia, hyperphosphatemia, hypo‐ or hypercalcemia and hyperkalaemia (Culp et al. 2016). In the present study, differences in Hb, RDW and ALB levels were observed among the CBC and serum biochemistry parameters. These mild increases in Hb, RDW and ALB observed in the URO/Non‐Ob, FIC/Non‐Ob and FIC/Ob groups, compared with the control group, may be associated with chronic inflammation or stress‐related physiological responses (He et al. 2022); however, their clinical relevance appears limited. Non‐specific clinical signs, including anorexia, reduced food and water intake, decreased general activity and vomiting, were frequently noted in the URO/Ob and FIC/Ob groups. These signs are likely attributable to bladder distension secondary to obstruction, associated pain and the consequent anorexia and dehydration (Astuty et al. 2020).

Ultrasonographic examination is a valuable diagnostic tool for identifying uroliths and/or obstructions in the urinary bladder and for differentiating the various causes of FLUTD (Vörös et al. 1997). Frequently observed ultrasonographic findings in cats with urethral obstruction include sediment formation in the urinary bladder, thickening of the bladder wall, hyperechoic pericystic fat and pericystic effusions (Nevins et al. 2015). Additionally, common ultrasonographic findings in non‐obstructive FLUTD include sediment accumulation in the urinary bladder and bladder wall thickening greater than 0.2 cm (Vörös et al. 1997; Gulersoy et al. 2022a; Gülersoy and Maden 2022). In the present study, ultrasonographic examination revealed acoustic shadowing and hyperechoic particles within the urinary bladder of cats in the URO/Ob and URO/Non‐Ob groups, whereas no significant ultrasonographic abnormalities were detected in the FIC/Ob group. Although the mean bladder wall thickness was greater in all FLUTD groups, compared with controls (0.14 cm), no statistically significant differences were identified among groups (*p* = 0.840). These findings are consistent with previous reports and highlight the limited ability of ultrasonography alone to distinguish between FLUTD subtypes with overlapping imaging features (Forrester and Towell 2015; Buffington and Chew 2017). Consequently, there is a clear need for adjunctive, non‐invasive biomarkers that reflect underlying bladder inflammation and tissue damage, thereby improving diagnostic discrimination, an objective addressed by the biomarker analyses presented below.

Neurotrophic growth factor (NGF) regulates the development and function of sensory neurons that transmit pain and also modulates the bladder reflex. NGF is produced by the smooth muscle and urothelial cells of the urinary bladder and can be detected in the urothelium, urine and serum (Westropp et al. 2003). NGF has been shown to play a key role in pain modulation and the pathogenesis of IC/BPS through its neuromodulatory effects. Accordingly, NGF has been proposed as a potential contributor to urinary bladder diseases, including IC/BPS in humans and FIC in cats (Lucon et al. 2010; Jayson and Seth 2013). A recent study further suggested that elevated serum NGF concentrations in cats with FIC may serve as a candidate biomarker for neurogenic inflammation and aid in the diagnosis of FIC. Moreover, NGF levels have been reported to be useful in the differential diagnosis of FIC and bacterial cystitis (BC) (Gulersoy et al. 2022a). As previously reported, decreased urinary NGF levels are associated with greater pain relief and favourable treatment outcomes. Additionally, loss of GAGs, linked to disruption of the bladder mucosal surface, contributes to the pathophysiology of IC/BPS in humans and FIC in cats. Notably, NGF concentrations were not elevated in the URO/Non‐Ob group in the present study. This finding suggests that NGF upregulation may be more specifically associated with FIC‐related neurogenic inflammation rather than with urolithiasis alone, thereby supporting its potential role in differentiating these conditions (Forrester and Towell 2015; Jones et al. 2021). Collectively, these observations contribute to a better understanding of the underlying mechanisms and may aid in the clinical monitoring of IC cases (Rubio‐Diaz et al. 2009; Jones et al. 2021; Gulersoy et al. 2022a). The GAG layer serves as a barrier that is crucial for preventing bacterial adhesion and providing physical protection to the urothelium. The decrease in the urothelial GAG layer results in increased bladder wall permeability (Buffington and Chew 2017). Chronic FIC cases are reported to be characterised by decreased urinary GAG secretion. This reduction in GAG concentrations facilitates deeper urine penetration into the bladder urothelium, which can initiate the inflammatory process. Damage to the GAG layer in the urinary bladder of cats is considered both a causative and consequential factor in the development of FIC (Lucon et al. 2010). A recent study (Gülersoy and Maden 2022) reported that urinary concentrations of GAG, IL‐12, NGF and tissue inhibitors of metalloproteinases‐2 (TIMP‐2) were higher in cats with FIC and BC, compared to healthy cats. In the present study, both serum and urine NGF levels were significantly elevated in the FIC/Ob, FIC/Non‐Ob and URO/Ob groups, compared to controls (*p* < 0.002 for urine; *p* < 0.0001 for serum). In contrast, GAG concentrations in both serum and urine were significantly reduced in the URO/Non‐Ob, FIC/Ob and FIC/Non‐Ob groups (*p* < 0.005 for urine; *p* < 0.0001 for serum). ROC analysis of the selected biomarkers indicated that NGF had limited utility for detecting obstruction in both serum and urine (AUC < 0.600), whereas GAG demonstrated acceptable diagnostic performance in obstructive cases (AUC > 0.600). In non‐obstructive cases, GAG was not a reliable marker (AUC < 0.600), whereas NGF provided valuable diagnostic information (AUC > 0.600; Tables 4 and 5). Overall, these findings suggest that NGF may be useful for diagnosing non‐obstructive conditions, while GAG is more informative in obstructive cases. However, no statistically significant difference was detected in serum and urine NGF levels for the differential diagnosis of FIC and URO cases. Although these results support the use of serum NGF concentration as a potential biomarker for diagnosing FIC cases (Gulersoy et al. 2022a), they also suggest that neurogenic inflammation may develop in cases of urolithiasis, likely due to urothelial damage caused by irritation from the uroliths. This finding is further supported by the comparative ROC analysis in the present study. Moreover, based on ROC analysis (Table 4; urine and serum GAG AUC > 0.600), urothelial damage appears to occur in obstructive cases regardless of aetiology (FIC or urolithiasis). These findings suggest that assessing GAG levels may be more valuable for monitoring treatment response or predicting prognosis than for initial diagnosis. Clinically, measuring serum and urine GAG concentrations could help evaluate the severity of urothelial injury and guide management decisions, even when the underlying cause of obstruction differs. In contrast, urine and serum NGF levels demonstrated acceptable diagnostic value in non‐obstructive cases (Table 5; AUC > 0.600), indicating that NGF warrants further investigation as a potential marker for elucidating the pathophysiology of cystitis (Bartges 2004; Culp et al. 2016; Gulersoy et al. 2022a). Accordingly, the elevated serum and urine NGF concentrations observed in the FIC and URO/Ob groups, compared to the healthy control group, are likely associated with the severity of neuroinflammation in the urinary bladder. (Gülersoy and Maden 2022). Although statistically significant differences were observed (Tables 4 and 5), the sensitivity and specificity values were modest, indicating limited diagnostic accuracy. Therefore, these biomarkers should not be considered standalone diagnostic tools but rather potential adjunctive markers that may support clinical evaluation.

A previous study reported serum and urine NGF cut‐off values of 7.74 and 7.31 ng/mL, respectively, which are lower than those observed in the present study. Because the earlier study used healthy cats as the comparison group, the higher cut‐off values here may reflect neuroinflammatory damage induced by uroliths, supporting the potential of NGF as a diagnostic and prognostic biomarker (Gulersoy et al. 2022a). Serum and urine GAG concentrations were decreased in all FIC groups, compared to healthy controls, suggesting increased urothelial permeability and confirming prior observations in chronic FIC cases (Buffington and Chew 2017). In contrast, GAG levels in the URO/Ob group were comparable to controls, suggesting that acute mechanical obstruction alone may not immediately disrupt the urothelial GAG layer. Unlike FIC, which is characterised by chronic inflammation and epithelial dysfunction, obstructive urolithiasis may primarily involve luminal blockage rather than direct or sustained mucosal damage. Therefore, GAG depletion may be more closely associated with chronic inflammatory or epithelial injury rather than obstruction per se. The reduced GAG levels observed in the FIC/Non‐Ob, FIC/Ob and URO/Non‐Ob groups may indicate underlying urothelial barrier dysfunction, increased permeability, and a predisposition to further epithelial injury (Lucon et al. 2010; Gülersoy and Maden 2022; Gulersoy et al. 2022a). Clinically, these findings suggest that measuring NGF and GAG levels could help identify and monitor urothelial damage, guide prognosis and inform management decisions in both FIC and urolith cases, regardless of obstruction (Lucon et al. 2010; Buffington and Chew 2017; Gulersoy et al. 2022a).

Limitations of this study include the absence of histopathological and cystoscopic assessments to directly evaluate the extent of bladder damage and inflammation, which could have provided more definitive pathological confirmation. Additionally, although the MEMO evaluation was used as an indicator of stress, objective biochemical markers of stress—such as serum or urinary cortisol—were not measured, which may have strengthened the assessment of stress responses. Another consideration is the relatively high cost and limited clinical availability of NGF and GAG assays, which may currently restrict their routine diagnostic application despite their potential utility. Future studies incorporating histopathology, stress hormone evaluation and cost‐effectiveness analyses will help provide a more comprehensive understanding of the diagnostic and clinical value of these biomarkers in FLUTD.

## 5 Conclusion

FLUTD cases commonly present with clinical signs such as dysuria, haematuria, stranguria, periuria and pollakiuria, with obstructive cases showing distinct symptom patterns, compared to FIC and non‐obstructive presentations. The present study demonstrates that serum and urine NGF concentrations are significantly elevated in FIC and provide moderate diagnostic accuracy in differentiating FIC from obstructive urolithiasis as supported by ROC analysis. Elevated NGF likely reflects bladder inflammation and neurogenic activity, consistent with its localisation in the bladder epithelium. Conversely, decreased urinary GAG concentrations, observed mainly in obstructive cases, correlate with urothelial damage and increased permeability. Together, NGF and GAG may serve as minimally invasive, adjunctive biomarkers that could aid in the differentiation of FLUTD subtypes when interpreted alongside clinical findings. Although the diagnostic performance of these biomarkers is modest, NGF and GAG may serve as supportive, minimally invasive markers that could complement conventional diagnostic approaches. Further studies with larger sample sizes are warranted to better define their clinical utility in differentiating FLUTD subtypes.

## Author Contributions

**Mehmet Maden, Ahmet Sarıkaya**: writing – original draft, writing – review and editing, investigation, data curation, software, formal analysis, conceptualisation, resources, project administration. **Mehmet Maden, Erdem Gülersoy**: writing – review and editing, validation, software, formal analysis, investigation, data curation. **Hacer Marangoz, Zafer Sayın**: writing – original draft, visualisation. **Mehmet Maden, Erdem Gülersoy, Ahmet Sarıkaya**: writing – original draft, visualisation. **Mehmet Maden, Hacer Marangoz, Zafer Sayın**: writing – review and editing, visualisation, resources.

## Funding

This study was supported by the Scientific Research Projects Coordinatorship of Selçuk University (SUBAPK, BAP, 23202030).

## Ethics Statement

This study was approved by the Selçuk University Animal Experiments Local Ethics Committee (Approval number: 2023/081). Also, all cat owners gave their consent before the commencement of the study. No experimental practices that will or can harm the animals or put their welfare at risk were done.

The study protocol was approved by the Faculty of Veterinary Medicine, Selçuk University Local Ethics Committee (Approval number: 2023/081). Informed consent was obtained from the owners of all cats enrolled in the study, and permissions were granted.

## Conflicts of Interest

The authors declare no conflicts of interest.

## Supporting information



Supporting File 1: vms370968‐sup‐0001‐figureS3.png

## Data Availability

The data that support the study findings are available from the authors upon request.

## References

[vms370968-bib-0001] Astuty, A. T. J. E. , I. Tjahajati , and W. S. Nugroho . 2020. “Detection of Feline Idiopathic Cystitis as the Cause of Feline Lower Urinary Tract Disease in Sleman Regency, Indonesia.” Veterinary World 13, no. 6: 1108–1112. 32801561 10.14202/vetworld.2020.1108-1112 PMC7396328

[vms370968-bib-0003] Barcelos, A. M. , K. McPeake , N. Affenzeller , and D. S. Mills . 2018. “Common Risk Factors for Urinary House Soiling (Periuria) in Cats and Its Differentiation: The Sensitivity and Specificity of Common Diagnostic Signs.” Frontiers in Veterinary Science 5: 108. 29892606 10.3389/fvets.2018.00108 PMC5985598

[vms370968-bib-0004] Bartges, J. W. 2004. “Diagnosis of Urinary Tract Infections.” Veterinary Clinics of North America Small Animal Practice 34, no. 4: 923–933. 15223209 10.1016/j.cvsm.2004.03.001

[vms370968-bib-0005] Bartges, J. W. 2011. “Urinary Saturation Testing.” In Nephrology and Urology of Small Animals, edited by J. Bartges and D. J. Polzin , 75–85. Wiley‐Blackwell.

[vms370968-bib-0006] Buffington, C. A. , and D. J. Chew . 2017. “Management of Non‐Obstructive Idiopathic/Interstitial Cystitis in Cats.” In BSAVA manual of Canine and Feline Nephrology and Urology, 3rd ed., edited by J. Elliott , G. F. Grauer , and J. Westropp , 317–327 BSAVA Library.

[vms370968-bib-0007] Buffington, C. A. , J. L. Westropp , D. J. Chew , and R. R. Bolus . 2006. “Clinical Evaluation of Multimodal Environmental Modification (MEMO) in the Management of Cats With Idiopathic Cystitis.” Journal of Feline Medicine and Surgery 8, no. 4: 261–268. 16616567 10.1016/j.jfms.2006.02.002 PMC10822542

[vms370968-bib-0008] Chew, D. J. , and C. A. T. Buffington . 2013. “Pandora Syndrome: It's More Than Just the bladder.” American Association of Feline Practitioners Conference. Dallas, Texas, September, no. 26: 75–83.

[vms370968-bib-0009] Culp, W. , C. Palm , C. Hsueh , et al. 2016. “Outcome in Cats With Benign Ureteral Obstructions Treated by Means of Ureteral Stenting Versus Ureterotomy.” Journal of the American Veterinary Medical Association 249: 1292–1300. 27875083 10.2460/javma.249.11.1292

[vms370968-bib-0010] Defauw, P. A. M. , I. Van de Maele , L. Duchateau , I. E. Polis , J. H. Saunders , and S. Daminet . 2011. “Risk Factors and Clinical Presentation of Cats With Feline Idiopathic Cystitis.” Journal of Feline Medicine and Surgery 13: 967–975. 22075439 10.1016/j.jfms.2011.08.001 PMC10832981

[vms370968-bib-0011] Dinler Ay, C. , G. E. Tuna , B. Ulutaş , and H. Voyvoda . 2021. “Clinicopathological Characteristics of Cats With Obstructive Lower Urinary Tract Disease in the Aydın Province (Turkey).” Kocatepe Veterinary Journal 14, no. 4: 474–481.

[vms370968-bib-0012] Dorsch, R. , C. Remer , C. Sauter‐Louis , and K. Hartmann . 2014. “Feline Lower Urinary Tract Disease in a German Cat Population: A Retrospective Analysis of Demographic Data, Causes and Clinical Signs.” Tierarztl Prax Ausg K Kleintiere Heimtiere 42, no. 4: 231–239. 25119631

[vms370968-bib-0013] Forrester, S. D. , and P. Roudebush . 2007. “Evidence‐Based Management of Feline Lower Urinary Tract Disease.” Veterinary Clinics of North America Small Animal Practice 37: 533–558. 17466755 10.1016/j.cvsm.2007.01.009

[vms370968-bib-0014] Forrester, S. D. , and T. L. Towell . 2015. “Feline Idiopathic Cystitis.” Veterinary Clinics of North America Small Animal Practice 45, no. 4: 783–806. 25813400 10.1016/j.cvsm.2015.02.007

[vms370968-bib-0015] Gomes, V. D. R. , P. C. Ariza , N. C. Borges , F. J. Schulz Jr. , and M. C. S. Fioravanti . 2018. “Risk Factors Associated With Feline Urolithiasis.” Veterinary Research Communications 42, no. 1: 87–94. 29340849 10.1007/s11259-018-9710-8

[vms370968-bib-0016] Gulersoy, E. , M. Maden , T. M. Parlak , and Z. Sayin . 2022a. “Comparative Evaluation of Selected Serum and Urine Biomarkers in Cats With Interstitial and Bacterial Cystitis.” Veterinary Clinical Pathology 52, no. 1: 79–87. 36345051 10.1111/vcp.13174

[vms370968-bib-0017] Gulersoy, E. , M. Maden , T. M. Parlak , and Z. Sayin . 2022b. “Diagnostic Effectiveness of Stress Biomarkers in Cats With Feline Interstitial and Bacterial Cystitis.” Veterinary Clinical Pathology 52, no. 1: 88–96. 36436835 10.1111/vcp.13173

[vms370968-bib-0018] Gülersoy, E. , and M. Maden . 2022. “Feline Intersitiel Cystitis—Pandora Syndrome /Kedilerin İntersitisyel Sistitisi‐Pandora Sendromu.” Turkiye Klinikleri Journal of Veterinary Sciences 13, no. 1: 20–29. 10.5336/vetsci.2021-83003.

[vms370968-bib-0019] He, C. , K. Fan , Z. Hao , N. Tang , G. Li , and S. Wang . 2022. “Prevalence, Risk Factors, Pathophysiology, Potential Biomarkers and Management of Feline Idiopathic Cystitis: An Update Review.” Frontiers in Veterinary Science 9: 900847. 35812890 10.3389/fvets.2022.900847 PMC9257190

[vms370968-bib-0020] Hesse, A. , H. Orzekowsky , M. Frenk , and R. Neiger . 2012. “Epidemiological Data of Urinary Stones in Cats Between 1981 and 2008.” Tierarztl Prax Ausg K Kleintiere Heimtiere 40, no. 2: 95–101. 22526813

[vms370968-bib-0021] Hosmer, D. W. , and S. Lemeshow . 2000. Applied Logistic Regression. 2nd ed. John Wiley & Sons, 160–164.

[vms370968-bib-0022] Jayson, S. , and M. Seth . 2013. “Feline Idiopathic Cystitis Management.” Vet Times 43: 1–18. https://www.vettimes.co.uk.

[vms370968-bib-0023] Jones, E. , C. Palmieri , M. Thompson , K. Jackson , and R. Allavena . 2021. “Feline Idiopathic Cystitis: Pathogenesis, Histopathology and Comparative Potential.” Journal of Comparative Pathology 185: 18–29. 34119228 10.1016/j.jcpa.2021.03.006

[vms370968-bib-0024] Kaul, E. , K. Hartmann , S. Reese , and R. Dorsch . 2020. “Recurrence Rate and Long‐Term Course of Cats With Feline Lower Urinary Tract Disease.” Journal of Feline Medicine and Surgery 22, no. 6: 544–556. 31322040 10.1177/1098612X19862887 PMC7252222

[vms370968-bib-0025] Kim, Y. , H. Kim , D. Pfeiffer , and D. Brodbelt . 2018. “Epidemiological Study of Feline Idiopathic Cystitis in Seoul, South Korea.” Journal of Feline Medicine and Surgery 20, no. 10: 913–921. 28967795 10.1177/1098612X17734067 PMC11129250

[vms370968-bib-0026] Lucon, M. , J. Dreyfuss , A. Silva , et al. 2010. “Glycosaminoglycans in Bladder Biopsies and in Urine as Possible Biomarkers for Interstitial Cystitis/Bladder Pain Syndrome.” Urology 76: S98.

[vms370968-bib-0027] Lulich, J. P. , and C. A. Osborne . 2009. “Changing Paradigms in the Diagnosis of Urolithiasis.” Veterinary Clinics of North America Small Animal Practice 39, no. 1: 127–141. 19038652 10.1016/j.cvsm.2008.10.005

[vms370968-bib-0028] Lund, H. S. , B. K. Saevik , W. F. Qystein , et al. 2016. “Risk Factors for Idiopathic Cystitis in Norwegian Cats: A Matched Case‐Control Study.” Journal of Feline Medicine and Surgery 18: 483–491. 26018550 10.1177/1098612X15587955 PMC11185226

[vms370968-bib-0029] Mattoo, T. K. , and J. D. Spencer . 2024. “Biomarkers for Urinary Tract Infection: Present and Future Perspectives.” Pediatric Nephrology 39, no. 10: 2833–2844. 38483594 10.1007/s00467-024-06321-9

[vms370968-bib-0030] Mayer, E. A. , and M. C. Bushnell . 2009. “Functional Pain Disorders: Time for a Paradigm Shift.” In Functional Pain Syndromes: Presentation and Pathophysiology, 531–566. IASP Press.

[vms370968-bib-0031] Nevins, R., Jr. , W. Mai , and E. Thomas . 2015. “Associations Between Ultrasound and Clinical Findings in 87 Cats With Urethral Obstruction.” Veterinary Radiology & Ultrasound 56, no. 4: 439–447. 10.1111/Vru.12259. 25850697

[vms370968-bib-0032] Nururrozi, A. , Y. Yanuartono , P. Sivananthan , and S. Indarjulianto . 2020. “Evaluation of Lower Urinary Tract Disease in the Yogyakarta Cat Population Indonesia.” Veterinary World 13, no. 6: 1182–1186. 32801571 10.14202/vetworld.2020.1182-1186 PMC7396331

[vms370968-bib-0033] Osborne, C. A. , J. P. Lulich , J. M. Kruger , L. K. Ulrich , and L. A. Koehler . 2009. “Analysis of 451,891 Canine Uroliths, Feline Uroliths, and Feline Urethral Plugs From 1981 to 2007: Perspectives From the Minnesota Urolith Center.” Veterinary Clinics of North America Small Animal Practice 39, no. 1: 183–197. 19038658 10.1016/j.cvsm.2008.09.011

[vms370968-bib-0034] Parys, M. , V. Yuzbasiyan‐Gurkan , and J. M. Kruger . 2018. “Serum Cytokine Profiling in Cats With Acute Idiopathic Cystitis.” Journal of Veterinary Internal Medicine 32: 274–279. 29356123 10.1111/jvim.15032 PMC5787166

[vms370968-bib-0035] Picavet, P. , E. Vasseur , M. Vasseur , M. Ponchon , and A. Strubel . 2019. “Urinary Tract Infection in Cats: Is This a Primary Problem?” Compendium: Continuing Education for Veterinarians 41, no. 7: 1–9.

[vms370968-bib-0036] Piyarungsri, K. , S. Tangtrongsup , N. Thitaram , P. Lekklar , and A. Kittinuntasilp . 2020. “Prevalence and Risk Factors of Feline Lower Urinary Tract Disease in Chiang Mai, Thailand.” Scientific Reports 10: 1–8. 31932653 10.1038/s41598-019-56968-w PMC6957472

[vms370968-bib-0037] Rubio‐Diaz, D. E. , M. E. Pozza , J. Dimitrakov , et al. 2009. “A Candidate Serum Biomarker for Bladder Pain Syndrome/Interstitial Cystitis.” Analyst 134, no. 6: 1133–1137. 19475139 10.1039/b901736d PMC3852842

[vms370968-bib-0038] Savary, K. C. , G. S. Price , and S. L. Vaden . 2000. “Hypercalcemia in Cats: A Retrospective Study of 71 Cases (1991‐1997).” Journal of Veterinary Internal Medicine 14: 184–189. 10772491 10.1892/0891-6640(2000)014<0184:hicars>2.3.co;2

[vms370968-bib-0039] Stella, J. L. , L. K. Lord , and C. A. Buffington . 2011. “Sickness Behaviors in Response to Unusual External Events in Healthy Cats and Cats With Feline Interstitial Cystitis.” Journal of the American Veterinary Medical Association 238, no. 1: 67–73. 21194324 10.2460/javma.238.1.67 PMC3852887

[vms370968-bib-0040] Vörös, K. , S. Wladár , A. Marsi , T. Vrabély , B. Fenyves , and T. Németh . 1997. “Ultrasonographic Study of Feline Lower Urinary Tract Diseases: 32 Cases.” Acta Veterinaria Hungarica 45: 387–395. 9557316

[vms370968-bib-0041] Westropp, J. L. , M. Delgado , and C. A. T. Buffington . 2019. “Chronic Lower Urinary Tract Signs in Cats: Current Understanding of Pathophysiology and Management.” The Veterinary Clinics of North America Small Animal Practice 49, no. 2: 187–209. 30736893 10.1016/j.cvsm.2018.11.001

[vms370968-bib-0042] Westropp, J. L. , and C. A. T. Buffington . 2004. “Feline Idiopathic Cystitis: Current Understanding of Pathophysiology and Management.” Veterinary Clinics of North America Small Animal Practice 34, no. 4: 1043–1055. 15223215 10.1016/j.cvsm.2004.03.002

[vms370968-bib-0043] Westropp, J. L. , K. A. Welk , and C. A. Buffington . 2003. “Small Adrenal Glands in Cats with Feline Interstitial Cystitis.” Urology Journal 170, no. 6: 2494–2497. 10.1097/01.ju.0000095566.63870.66 14634458

