# Supplementary figures and images for: Serum and Urine Nerve Growth Factor and Glycosaminoglycan Levels in Obstructive and Non‐Obstructive Feline Urolithiasis and Interstitial Cystitis
Source: Vet Med Sci. 2026 May 20;12(3):e70968. doi: 10.1002/vms3.70968 (PMC13240338; doi:10.1002/vms3.70968)

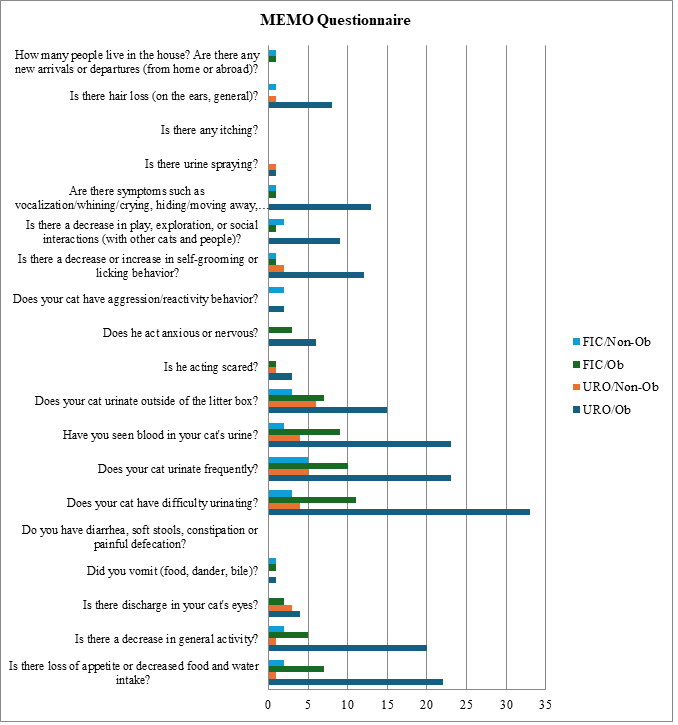

Supplement: Supplementary file 1 — Supporting File 1: vms370968‐sup‐0001‐figureS3.png [file VMS3-12-e70968-s001.png]
